# Expression and Prognostic Value of MCM Family Genes in Osteosarcoma

**DOI:** 10.3389/fmolb.2021.668402

**Published:** 2021-06-22

**Authors:** Jian Zhou, Mingyong Wang, Zhen Zhou, Wanchun Wang, Juan Duan, Gen Wu

**Affiliations:** ^1^ Department of Orthopedics, The Second Xiangya Hospital, Central South University, Changsha, China; ^2^ College of Pharmaceutical Sciences, Soochow University, Suzhou, China; ^3^ Institute of Osteoporosis Diagnosis and Treatments of Soochow University, Suzhou, China; ^4^ Menzies Institute for Medical Research, University of Tasmania, Hobart, TAS, Australia; ^5^ Department of Geriatrics, The Second Xiangya Hospital, Central South University, Changsha, China

**Keywords:** MCMs, sarcoma, expression, prognosis, DNA methylation

## Abstract

We performed a detailed cancer VS normal analysis to explore the expression and prognostic value of minichromosome maintenance (MCM) proteinsin human sarcoma. The mRNA expression levels of the MCM family genes in sarcoma were analyzed using data from ONCOMINE, GEPIA and CCLE databases. KEGG database was used to analyze the function of MCM2–7 complex in DNA replication and cell cycle. QRT-PCR and western blot were used to confirm the differential expression of key MCMs in osteosarcoma cell lines. Cell Counting Kit-8 and flow cytometry method were used to detect the cell proliferation and apoptosis of hFOB1.19 cells. The results showed that MCM1–7 and MCM10 were all upregulated in sarcoma in ONCOMINE database. MCM2, and MCM4–7 were highly expressed in sarcoma in GEPIA database. Moreover, all these ten factors were highly expressed in sarcoma cell lines. Furthermore, we analyzed the prognostic value of MCMs for sarcoma in GEPIA and found that MCM2, MCM3, MCM4, and MCM10 are prognostic biomarkers for human sarcoma. Analysis results using KEGG datasets showed that MCM4 and MCM6–7 constituted a core structure of MCM2-7 hexamers. We found that AzadC treatment and overexpression of MCM4 significantly promoted hFOB1.19 cell proliferation and inhibited apoptosis. The present study implied that MCM2–4 and 10 are potential biomarkers for the prognosis of sarcoma. The prognostic role of MCM4 may be attributable to the change in its DNA methylation patterns.

## Introduction

Sarcomas are malignant tumors derived from mesenchymal tissues (including connective tissue and muscle), and they mostly occur in the skin, subcutaneous, periosteum and both ends of long bones. Sarcomas can be divided into soft tissue sarcoma and osteosarcoma. Osteosarcoma is more common in adolescents and occurs in the metaphysis of the extremities, especially the distal femur and proximal tibia. Common symptoms of osteosarcoma include pain, swelling, numbness, varicose veins, and even pathological fractures. Osteosarcoma is highly malignant, grows rapidly, and often spreads to the lungs through blood. Some sarcomas, in contrast, are more common in the elderly, such as liposarcoma and leiomyosarcoma, of which the clinical manifestations are often non-specific and are often related to the degree of the malignancy. Soft tissue sarcomas account for about 0.8% of human malignant tumors, with the annual incidence rate of about 2.38/100,000 in China and increasing significantly with age. The most common site of soft tissue sarcomas is limb, accounting for 53%, followed by retroperitoneum (19%), trunk (12%), head and neck (11%). The most common primary bone malignant tumor is classic osteosarcoma, which accounts for about 0.2% of human malignant tumors. The most common sites of incidence are the distal femur and proximal tibia, followed by In the proximal humerus, osteosarcomas in these three locations account for approximately 85% of all osteosarcomas. Now, sarcomas are divided into two categories at the molecular level: 1) genetically complex, high mutation burden, and complex karyotype; and 2) genetically simple, with a single disease-specific translocation, mutation, or amplification in a relatively static genome background, mutation or amplification. This histological and molecular heterogeneity makes sarcomas particularly difficult to diagnose. Therefore, new biomarkers are needed as prognostic indicators to guide individualized treatment and improve patients’ prognosis (Dancsok et al., 2017).

Minichromosome maintenance proteins (MCMs) comprised ten proteins, which play essential roles in DNA replication and cell cycle progression (Maiorano et al., 2006; Li et al., 2019). They were first discovered in *Saccharomyces cerevisiae* and identified as essential factors for the maintenance of extra-chromosomal DNA (Ishimi, 2018). MCM1 influences the process of cell cycle, growth, differentiation, and apoptosis by regulating the activation of multiple genes. It belongs to MADS box transcription factor family (Pramila et al., 2002). The MCM2-7 form a heterohexameric complex that serves as a DNA duplicating helicase, which unwinds the DNA duplex template during DNA replication (Fei and Xu, 2018; Suzuki et al., 2019). MCM8 and nine were reported to make up the CMG complex with Cdt1 and GINS. (Griffin and Trakselis, 2019). MCM10 plays an important role in the initiation of DNA replication and elongation (Baxley and Bielinsky, 2017). As same as many DNA replication proteins, MCM proteins were reported to play an important role in cancer development (Yu et al., 2020). MCMs has been detected that were overexpressed in multiple cancer tissues and carcinoma cell lines, such as lymphomas (Marnerides et al., 2011; Rusiniak et al., 2012), brain tumors (Winther and Torp, 2017; Cai et al., 2018), gastrointestinal tract tumors (Giaginis et al., 2009; Shomori et al., 2010; Peng et al., 2016), breast cancer (Wojnar et al., 2011), prostate cancer (Stewart et al., 2017), renal cell carcinoma (Zhong et al., 2017), and lung squamous cell carcinoma (Wu et al., 2018). However, the role of MCM family members in the development of sarcoma has not been fully understood, and its prognostic value for sarcoma is still unknown. In this present study, we used database research and bioinformatic analysis to assess the expression of MCMs in sarcoma and analyze its prognostic value for sarcoma.

## Methods

### Oncomine Analysis

Oncomine is currently the world’s largest cancer gene chip database and integrated data mining platform. It aims to mine cancer genetic information. It integrates RNA and DNA-seq data from GEO, TCGA and published literature sources, and has the most complete cancer mutation spectrum, gene expression data and related clinical information. It can be used to discover new biomarkers and new therapeutic targets.

With using Oncomine datasets, we performed multiple expression analyses of MCMs in sarcoma and normal samples. The *p* value was generated by using a Students’ t-test. We defined the cut-off of *p*-value and fold change as 0.01 and 2, respectively. It was also used to find the co-expression genes of MCMs in sarcoma.

### Gene Expression Profile Interactive Analysis Dataset

GEPIA (Gene Expression Profile Interactive Analysis) is a web-based tool for delivering fast and customizable functionalities based on TCGA and GTEx data. In our study, Data from the GEPIA datasets were used to analyze the different expression levels of MCMs in sarcoma and normal tissues. We also analyzed the correction between MCMs in sarcoma by using the GEPIA dataset. In order to explore the prognostic value of MCMs in sarcoma patients, GEPIA was also used to analyze the association of the expression levels of MCMs with the OS and DFS.

### Cancer Cell Line Encyclopedia Dataset

CCLE (Cancer Cell Line Encyclopedia) is a tumor genomics research project led by the Broad Institute. It collects and sorts out the omics data of 1,457 cell lines. We analyzed the expression levels of the ten MCMs in sarcoma cell lines, by using CCLE dataset.

### Kyoto Encyclopedia of Genes and Genomes Dataset

KEGG (Kyoto Encyclopedia of Genes and Genomes) is a practical database resource for understanding advanced functions and biological systems (such as cells, organisms, and ecosystems), genome sequencing and other high-throughput experimental technologies generated from molecular level information, especially large molecular data sets. In our study, we used data from the KEGG to analyze the functional role of MCM2–7 complex in DNA replication and cell cycle.

### CpG Island Prediction

We obtained the human MCM4 (ENSG00000104738) sequence from Ensembl genome browser (http://asia.ensembl.org/index.html). We used MethPrimer software (http://www.urogene.org/methprimer) to analyze CpG islands, and to detect the first exon sequence. We predict the candidate transcription factor by JASPAR (http://jaspar.binf.ku.dk/).

### Cell Culture

We purchase hFOB1.19 cell line in Chinese Science Institute (Shanghai, China). hFOB1.19 cells were cultured in Roswell Park Memorial Institute (RPMI) 1,640 medium (HyClone, Logan, UT, United States), containing 10% fetal bovine serum (FBS) (Sijiqing, Hangzhou, China).

### Total RNA Isolation and qRT-PCR

We isolate RNA from 100 mg of tissue (liquid nitrogen grounding method performed before RNA extraction) and 2 × 106 cells by TRIzol Reagent (Invitrogen, Carlsbad, CA, United States). We performed RNA qualification and quantification using Biotek (Winooski, VT, United States). A total of 2 μg RNA was reverse transcribed to cDNA with Superscript III Reverse Transcriptase (Invitrogen). Quantitative real-time PCRs (qRT-PCRs) were conducted in an ABI StepOnePlus instrument, with the SYBR (TaKaRa) system, and a thermal profile of 40 cycles of 95°C for 10 s and 58°C for 30 s. All of the results were standardized to the expression level of the housekeeping gene, β-actin. Relative mRNA expression levels were calculated using 2^−△△^ CT.

### Western Blot

We washed the monolayer cells with 1× PBS and extracted the proteins by using RIPA lysis buffer. All the specimens were centrifuged (10000 g, 4°C for 10 min). Protein concentration was measured using BCA protein assay kit (Beyotime). Proteins were resolved by 10% SDS-PAGE and then transferred to PVDF membranes which was blocked using 5% non-fat milk in 1× TBS mixed with Tween-20. After that, the membrane was incubated overnight with anti-MCM2/4 antibody (1:2000, abcam) at 4°C. The PVDF membrane was washed using 1 × TBS-T for 15 min for 3 times. Secondary anti-rabbit IgG antibody was used to incubate (1:10,000, biosharp) for 1 h. Electrochemiluminescence was added to PVDF membrane and the membrane was exposed on an X-ray film.

### Cell Proliferation Assay

We analyzed cell proliferation by the Cell Counting Kit-8 (Dojindo). After 2 days, hFOB1.19 cells were inducible. AzadC-treated cells were then incubated for another 24 h. A BioTek microplate reader was used to measure the optical density of each group.

### Cell Apoptosis Assay

We analyzed the hFOB1.19 cell apoptosis using flow cytometry method (FCM). We collected the cells 2 days after treatment with AzadC, washed with PBS, and suspended in 500 μL binding buffer. The cells were incubated with annexin V at room temperature for 10 min and stained with PI, and then analyzed by FCM for relative quantitative apoptosis.

### Statistics

The data were presented as mean ± SD and statistical differences were determined with Student’s t test. In the presented study, the experiments conducted have been repeated for 3 times. The representative experiments were shown in results. Significant differences were defined at *p* < 0.05.

## Results

### Transcriptional Levels of Minichromosome Maintenance in Patients With Sarcoma

**FIGURE 1:**
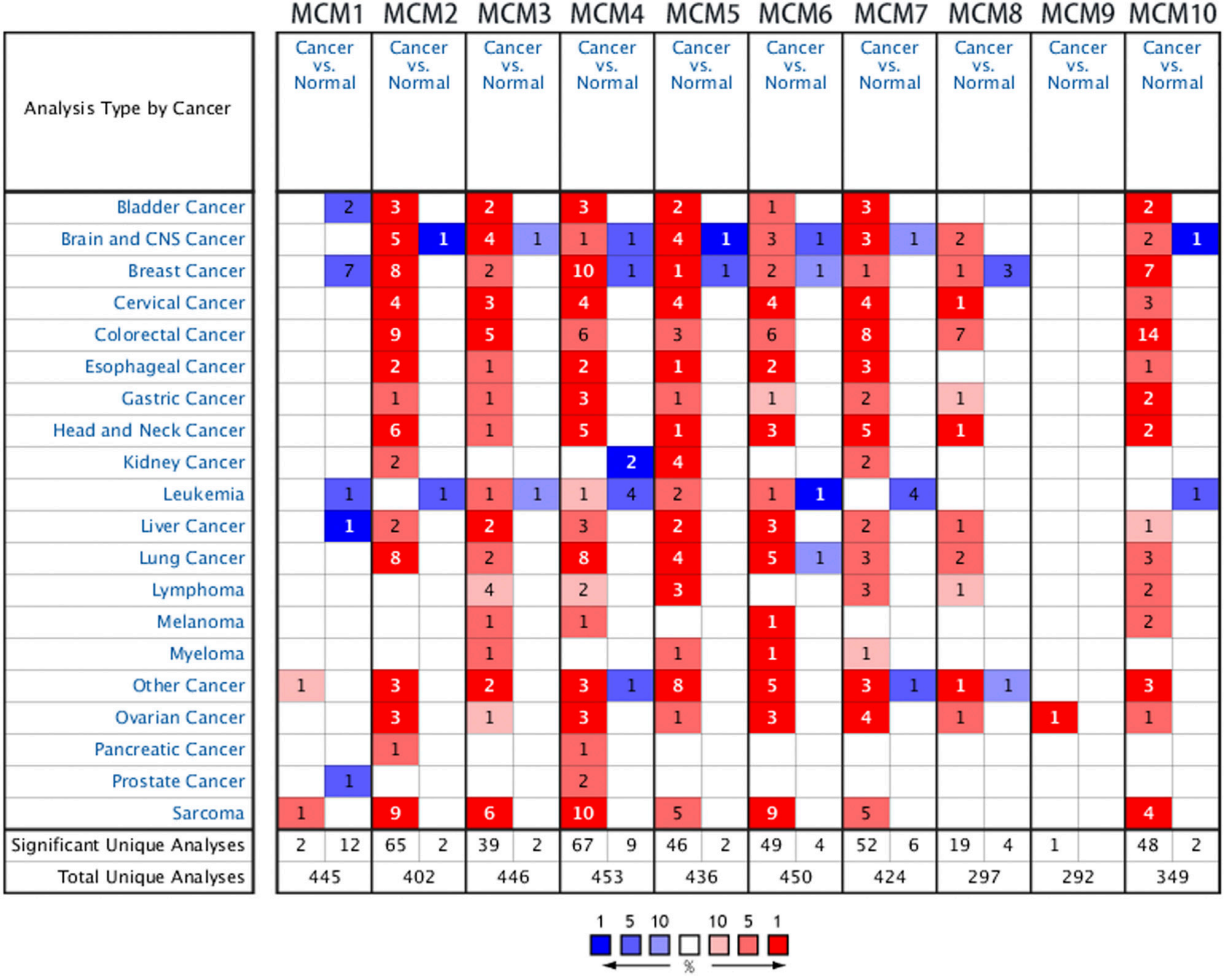
The transcription levels of MCM factors in different types of cancers.

Previous studies have identified ten MCM factors in eukaryotic cells and archaea (Maiorano et al., 2006). In the present study, we used ONCOMINE database to compare the transcription levels of MCMs in cancer and normal tissues. The results showed that MCMs were generally upregulated in various of tumors. In sarcoma, most of MCM members were highly expressed in cancer tissues, except for MCM8 and MCM9 (Figure 1). The mRNA expression levels of MCMs were showed in Table 1. In the datasets of Detwiller Sarcoma (Detwiller et al., 2005), compared with normal tissues, MCM1 was overexpressed in leiomyosarcoma with a fold change of 2.063 (Table 1).

**TABLE 1:** The significant changes of MCMs expression in subgroups of sarcoma.

Gene ID	Types of sarcoma vs. Normal	Fold change	*p* Value	*t* Test	Renfrences
MCM1	Leiomyosarcoma vs. Normal	2.063	9.03E-5	4.752	Detwiller sarcoma
MCM2	Uterine corpus leiomyosarcoma vs. Normal	26.250	3.47E-7	12.037	Quade uterus
	Pleomorphic liposarcoma vs. Normal	3.538	5.71E-14	12.932	Barretina sarcoma
	Myxofibrosarcoma vs. Normal	3.819	3.38E-16	14.472	Barretina sarcoma
	Leiomyosarcoma vs. Normal	4.437	1.15E-13	11.848	Barretina sarcoma
	Myxoid/Round cell liposarcoma vs. Normal	3.012	7.51E-13	13.922	Barretina sarcoma
	Dedifferentiated liposarcoma vs. Normal	2.539	8.57E-13	10.845	Barretina sarcoma
	Leiomyosarcoma vs. Normal	7.904	1.54E-7	8.860	Detwiller sarcoma
	Fibrosarcoma vs. Normal	4.867	2.06E-6	6.741	Detwiller sarcoma
	Malignant fibrous histiocytoma vs. Normal	4.180	1.05E-6	6.440	Detwiller sarcoma
MCM3	Pleomorphic liposarcoma vs. Normal	2.316	2.00E-13	12.368	Barretina sarcoma
	Myxoid/Round cell liposarcoma vs. Normal	2.769	9.19E-14	13.442	Barretina sarcoma
	Myxofibrosarcoma vs. Normal	2.122	1.02E-11	9.391	Barretina sarcoma
	Leiomyosarcoma vs. Normal	2.212	9.93E-10	8.245	Barretina sarcoma
	Fibrosarcoma vs. Normal	2.979	1.98E-7	8.423	Detwiller sarcoma
	Synovial sarcoma vs. Normal	2.167	4.19E-5	6.349	Detwiller sarcoma
MCM4	Pleomorphic liposarcoma vs. Normal	3.142	3.36E-15	14.259	Barretina sarcoma
	Myxofibrosarcoma vs. Normal	3.455	1.12E-15	12.861	Barretina sarcoma
	Dedifferentiated liposarcoma vs. Normal	2.195	6.46E-14	10.370	Barretina sarcoma
	Leiomyosarcoma vs. Normal	3.042	9.01E-11	9.360	Barretina sarcoma
	Myxoid/Round cell liposarcoma vs. Normal	2.227	3.15E-10	9.407	Barretina sarcoma
	Pleomorphic liposarcoma vs. Normal	13.735	1.34E-8	10.545	Detwiller sarcoma
	Fibrosarcoma vs. Normal	10.126	2.41E-8	8.507	Detwiller sarcoma
	Malignant fibrous histiocytoma vs. Normal	10.011	1.50E-8	8.331	Detwiller sarcoma
	Synovial sarcoma vs. Normal	3.401	5.28E-7	7.400	Detwiller sarcoma
	Leiomyosarcoma vs. Normal	8.153	2.50E-6	6.623	Detwiller sarcoma
MCM5	Pleomorphic liposarcoma vs. Normal	3.772	8.63E-10	10.061	Barretina sarcoma
	Leiomyosarcoma vs. Normal	3.538	2.41E-9	8.260	Barretina sarcoma
	Myxoid/Round cell liposarcoma vs. Normal	2.817	7.49E-8	8.303	Barretina sarcoma
	Dedifferentiated liposarcoma vs. Normal	2.536	8.54E-7	8.457	Barretina sarcoma
	Malignant fibrous histiocytoma vs. Normal	4.703	8.32E-5	4.561	Detwiller sarcoma
MCM6	Leiomyosarcoma vs. Normal	5.847	3.58E-7	8.247	Detwiller sarcoma
	Fibrosarcoma vs. Normal	5.500	5.30E-7	7.678	Detwiller sarcoma
	Malignant fibrous histiocytoma vs. Normal	4.896	7.34E-7	6.930	Detwiller sarcoma
	Synovial sarcoma vs. Normal	4.175	2.67E-5	6.725	Detwiller sarcoma
	Pleomorphic liposarcoma vs. Normal	3.300	3.76E-12	10.849	Barretina sarcoma
	Myxofibrosarcoma vs. Normal	3.234	3.06E-15	12.962	Barretina sarcoma
	Leiomyosarcoma vs. Normal	3.034	1.41E-12	10.712	Barretina sarcoma
	Dedifferentiated liposarcoma vs. Normal	2.076	2.14E-10	10.804	Barretina sarcoma
	Myxoid/Round cell liposarcoma vs. Normal	2.141	2.91E-10	9.875	Barretina sarcoma
MCM7	Myxoid/Round cell liposarcoma vs. Normal	3.047	1.35E-10	12.092	Barretina sarcoma
	Pleomorphic liposarcoma vs. Normal	2.349	1.27E-8	9.695	Barretina sarcoma
	Leiomyosarcoma vs. Normal	2.288	9.17E-9	8.526	Barretina sarcoma
	Myxofibrosarcoma vs. Normal	2.339	9.65E-9	9.552	Barretina sarcoma
	Fibrosarcoma vs. Normal	2.236	3.44E-5	5.259	Detwiller sarcoma
MCM8	NA	NA	NA	NA	NA
MCM9	NA	NA	NA	NA	NA
MCM10	Round cell liposarcoma vs. Normal	7.893	2.81E-7	8.065	Detwiller sarcoma
	Malignant fibrous histiocytoma vs. Normal	7.758	5.16E-8	7.751	Detwiller sarcoma
	Synovial sarcoma vs. Normal	5.892	7.40E-6	6.531	Detwiller sarcoma
	Fibrosarcoma vs. Normal	9.258	1.26E-5	6.289	Detwiller sarcoma

Quade Uterus datasets (Quade et al., 2004) showed that MCM2 was overexpressed in uterine corpus leiomyosarcoma with a fold change of 26.250. The datasets of Barretina Sarcoma (Barretina et al., 2010) also showed the increased expression of MCM2. The fold change of MCM2 in patients with pleomorphic liposarcoma, myxofibrosarcoma, leiomyosarcoma, myxoid/round cell liposarcoma and dedifferentiated liposarcoma was 3.538, 3.819, 4.437, 3.012, and 2.539, respectively. Using datasets of Detwiller Sarcoma, MCM2 was also found to be overexpressed in leiomyosarcoma (fold change = 7.904), fibrosarcoma (fold change = 4.867) and malignant fibrous histiocytoma (fold change = 4.180) compared with normal samples (Table 1).

Using Barretina Sarcoma’s datasets, MCM3 was found to be overexpressed in pleomorphic liposarcoma (fold change = 2.316), myxoid/round cell liposarcoma (fold change = 2.769), myxofibrosarcoma (fold change = 2.122) and leiomyosarcoma (fold change = 2.212) when compared with normal samples. MCM3 was also overexpressed in fibrosarcoma with a fold change of 2.979 and synovial sarcoma with a fold change of 2.167, reported in Detwiller Sarcoma (Table 1).

Using datasets of Barretina Sarcoma, compared with normal samples, MCM4 was overexpressed in pleomorphic liposarcoma, myxofibrosarcoma, dedifferentiated liposarcoma, leiomyosarcoma and myxoid/round cell liposarcoma, with the fold change of 3.142, 3.455, 2.195, 3.042, and 2.227 respectively. In Detwiller Sarcoma’s datasets, MCM4 was also showed to be overexpressed in pleomorphic liposarcoma (fold change = 13.735), fibrosarcoma (fold change = 10.126), malignant fibrous histiocytoma (fold change = 10.011), synovial sarcoma (fold change = 3.401) and leiomyosarcoma (fold change = 8.153), compared with normal samples (Table 1).

Analysis using Barretina Sarcoma’s datasets found a higher expression of MCM5 in pleomorphic liposarcoma (fold change = 3.772), leiomyosarcoma (fold change = 3.538), myxoid/round cell liposarcoma (fold change = 2.817) and dedifferentiated liposarcoma (fold change = 2.536), when compared with normal samples. Using Detwiller Sarcoma’s datasets, MCM5 was also found to be overexpressed in malignant fibrous histiocytoma (fold change = 4.703) compared with normal samples. (Table 1).

Overexpression of MCM6 in sarcoma was also found in both datasets. In Detwiller Sarcoma’s datasets, MCM6 was overexpressed in leiomyosarcoma (fold change = 5.847), fibrosarcoma (fold change = 5.500), malignant fibrous histiocytoma (fold change = 4.896) and synovial sarcoma (fold change = 4.175). In Barretina Sarcoma’s datasets, MCM6 was overexpressed in pleomorphic liposarcoma (fold change = 3.300), myxofibrosarcoma (fold change = 3.234), leiomyosarcoma (fold change = 3.034), dedifferentiated liposarcoma (fold change = 2.076) and myxoid/round cell liposarcoma (fold change = 2.141) compared with normal samples (Table 1).

Analyses using these two datasets also showed the overexpression of MCM7 in sarcoma. Using Barretina Sarcoma’s datasets, the results showed that MCM7 was higher expressed in myxoid/round cell liposarcoma (fold change = 3.047), pleomorphic liposarcoma (fold change = 2.349), leiomyosarcoma (fold change = 2.288) and myxofibrosarcoma (fold change = 2.339) compared with normal samples. Using Detwiller Sarcoma’s datasets, the results showed the overexpression of MCM7 in fibrosarcoma (fold change = 2.236) compared with normal samples (Table 1).

Overexpression of MCM10 was found in the analysis using Detwiller Sarcoma’s datasets. The MCM10 fold change of patients with round cell liposarcoma, Malignant fibrous histiocytoma, synovial Sarcoma and fibrosarcoma was 7.893, 7.758, 5.892, and 9.258, respectively (Table 1).

### Association Between Minichromosome Maintenance mRNA Levels and Clinicopathological Parameters in Patients With Sarcoma

**FIGURE 2:**
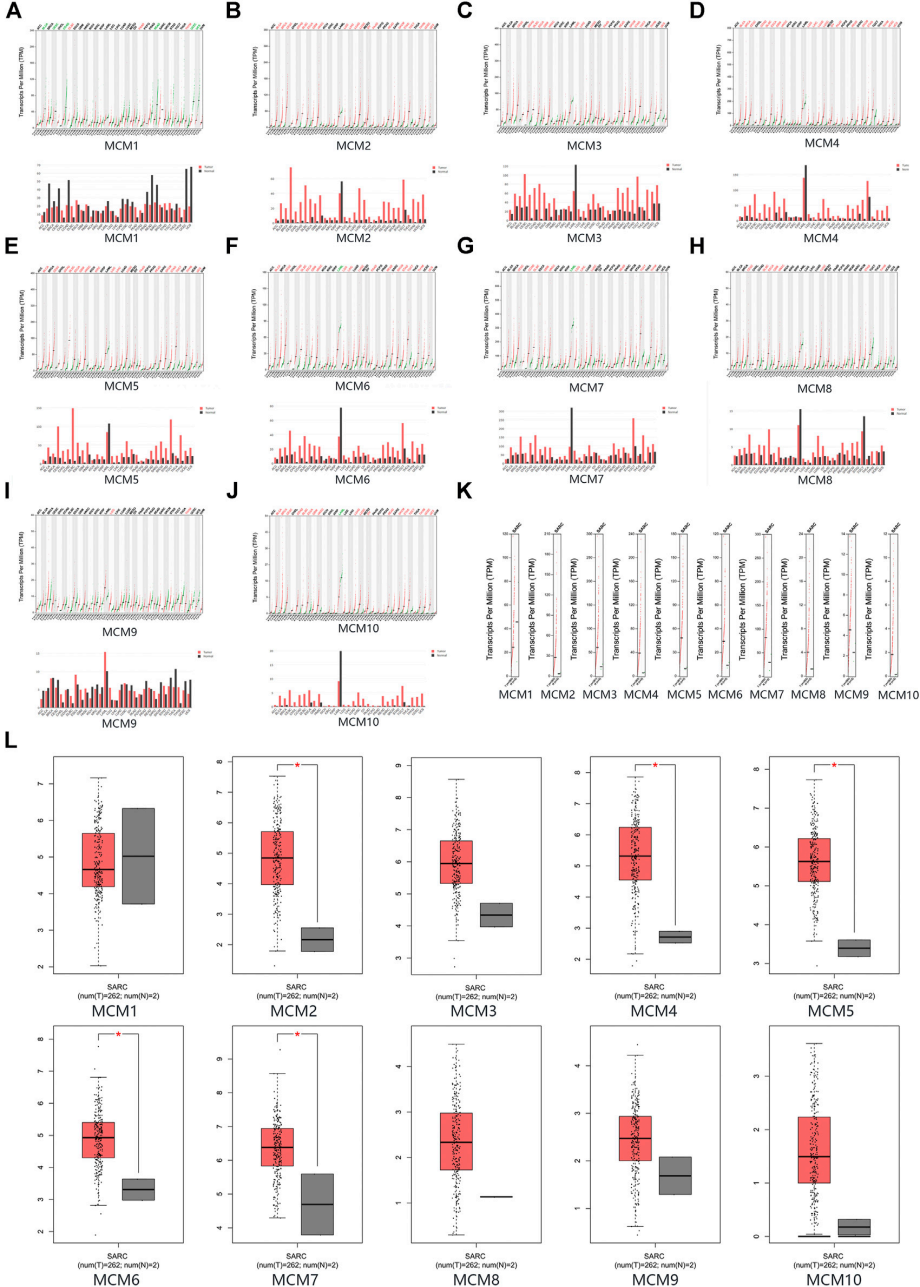
The expression of MCMs in sarcoma. **(A–J)** The expression of MCM1-10 in pan-cancer. **(K–L)** The expression of MCMs in sarcoma.

The mRNA expression levels of MCM factors in sarcoma and normal tissues were compared using GEPIA datasets. We found that except for MCM1, all other MCM factors had higher expression levels in sarcoma than in normal tissues (*p* < 0.05 for MCM2, MCM4, MCM5, MCM6 and MCM7) (Figures 2A–L).

### Minichromosome Maintenance Expression in Sarcoma Cell Lines

**FIGURE 3:**
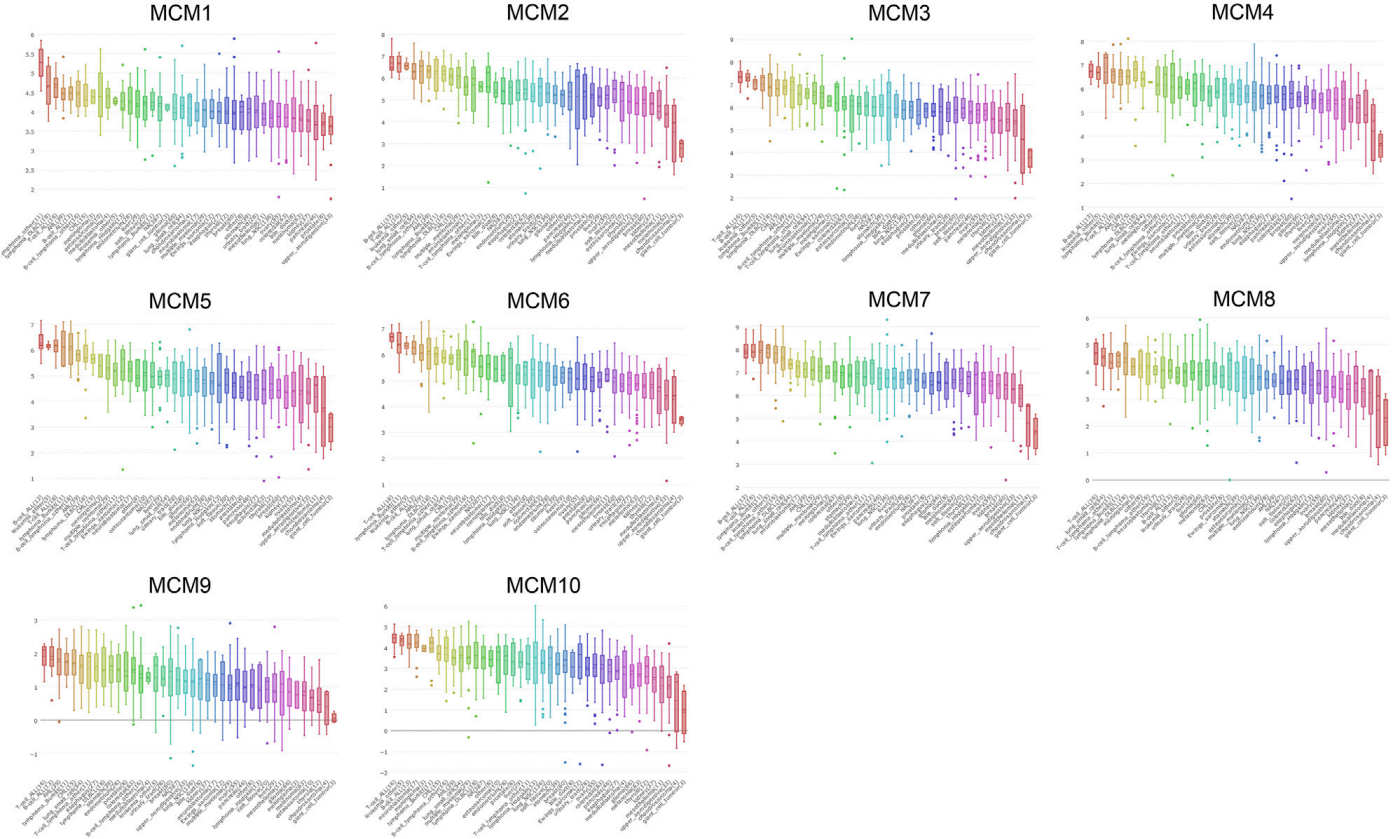
The expression of MCMs sarcoma cell lines.

CCLE was used to expand the detailed annotation process of the preclinical human cancer models. We found that the ten MCM family members were all highly expressed in sarcoma cell lines (Figure 3).

### The Prognostic Values of Minichromosome Maintenance in Sarcoma

**FIGURE 4:**
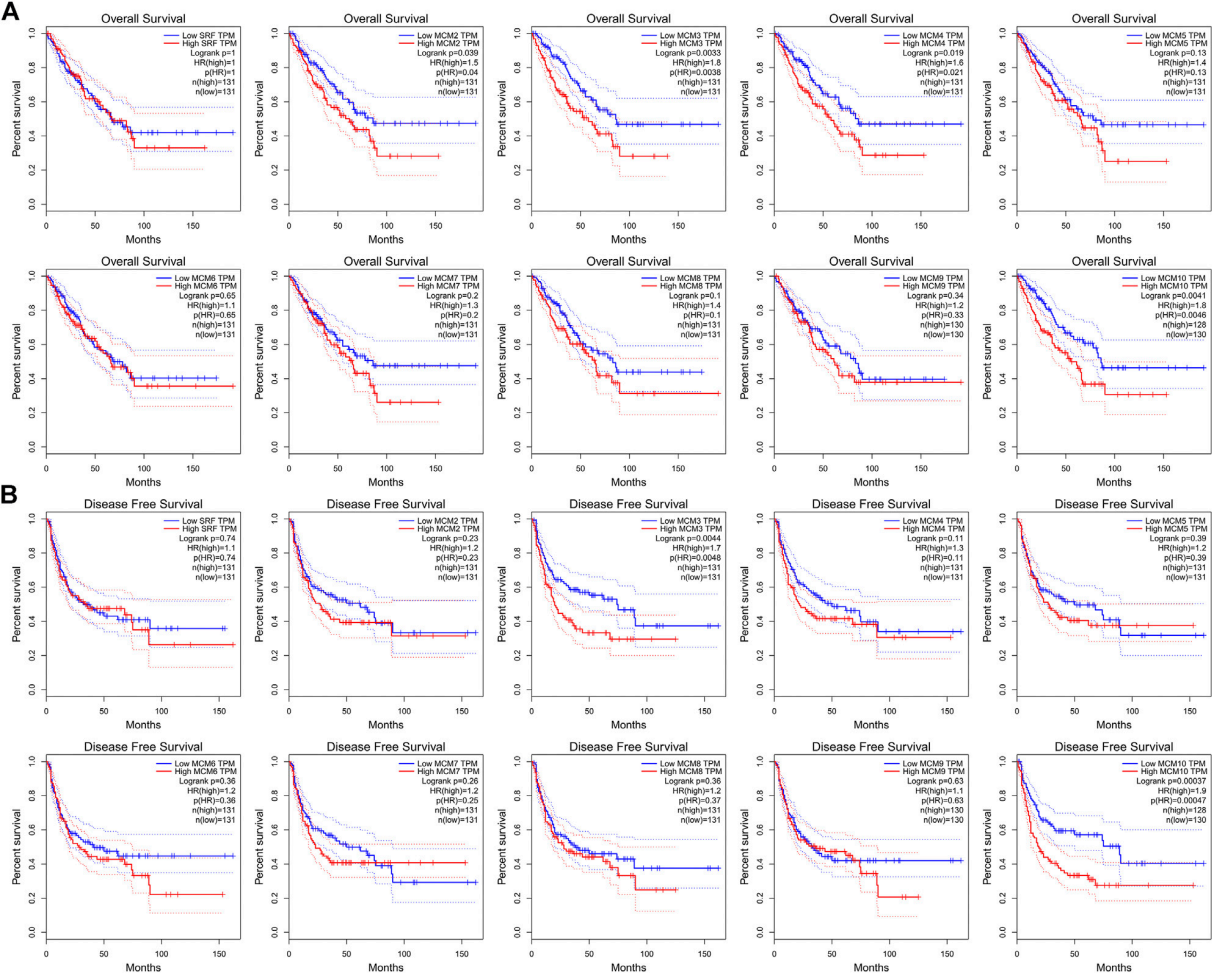
The prognostic value of MCMs in patients with sarcoma. **(A)** The association between MCM genes and overall survival in patients with sarcoma. **(B)** The association between MCM genes and disease-free survival in patients with sarcoma. HR, hazard ratio; TPM, Transaction per million.

We investigated the prognostic role of the ten MCM factors in sarcoma by using the GEPIA online service. The results showed that high levels of MCM3 and MCM10 mRNA significantly decreased the over survival (OS) (*p* < 0.05) and disease-free survival (DFS) (*p* < 0.05) of sarcoma patients (Figures 4A,B). Moreover, high levels of MCM2 and MCM4 mRNA significantly decreased the OS (*p* < 0.05) of sarcoma patients (Figure 4A). The mRNA expression levels of other MCM factors had no statistically significant effect on OS and DFS in patients with sarcoma (Figures 4A,B). Therefore, MCM2, MCM3, MCM4, and MCM10 were four potential biomarkers for the prognosis of sarcoma and a higher expression indicates worse outcomes.

### Kyoto Encyclopedia of Genes and Genomes Analysis and Venn Diagram Analysis

**FIGURE 5:**
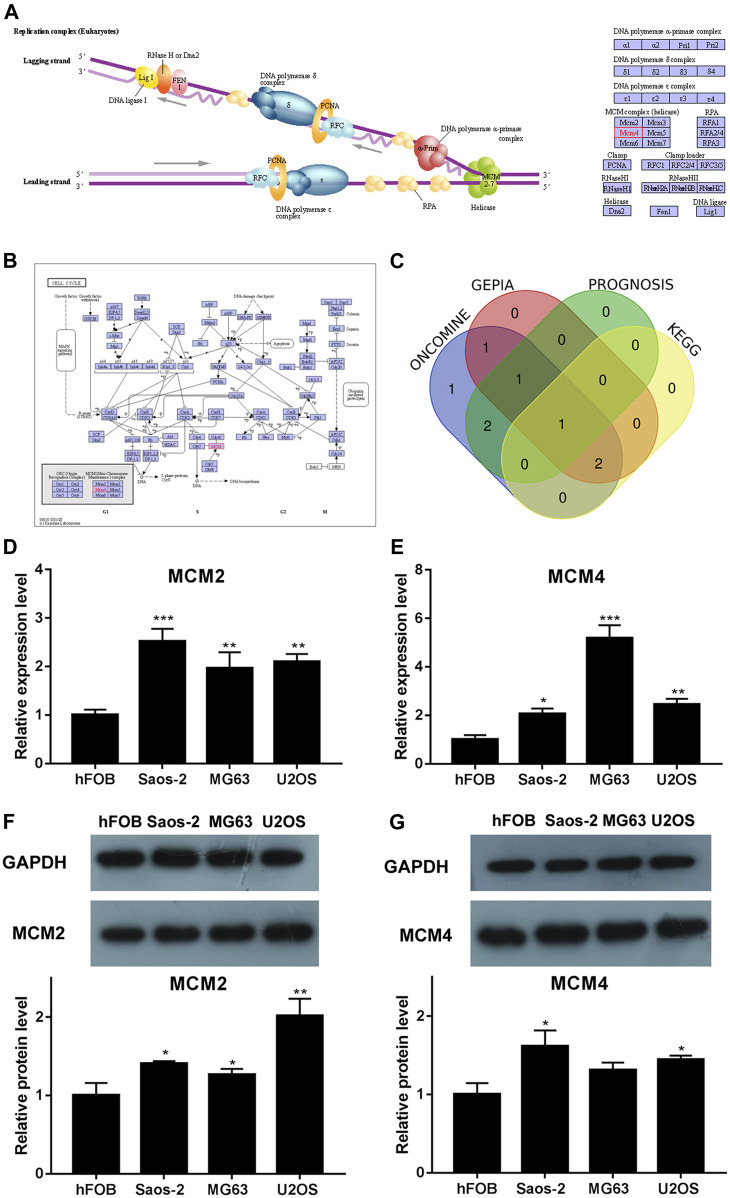
KEGG analysis of MCMs, crosscheck of analysis and differential expression of MCM2 and MCM4 in osteosarcoma cell lines. **(A)** DNA replication and **(B)** cell cycle pathway regulated by the MCM alteration in sarcoma (KEGG). **(C)** Crosscheck of the results of ONCOMIN, GEPIA, Prognosis and KEGG analysis. Elevated expression of **(D)** MCM2 and **(E)** MCM4 detected in osteosarcoma cell lines using qRT-PCR. Up regulated expression of **(F)** MCM2 and **(G)** MCM4 detected in osteosarcoma cell lines using western blot, *p < 0.05, **p < 0.01, ***p < 0.001.

We analyzed the pathways related to the function changes of MCMs by using KEGG datasets. The results showed that MCM2–7 proteins formed a heterohexamer complex and participated in the initial step of DNA synthesis (Figure 5A). The cell cycle pathway was involved in the tumorigenesis and pathogenesis of sarcoma (Figure 5B). A venn diagram was used to show the relationship between ONCOMINE, GEPIA, PROGNOSIS biomarker and KEGG datasets (Figure 5C). According to the results, we found that MCM2 and MCM4 were elevated expressed in ONCOMINE and GEPIA datasets with prognostic values, so the key MCM family genes including MCM2 and MCM4 were chosen to be confirmed in sarcoma cell lines (Figures 5D–G). Previous study indicated that MCM4 and MCM6-7 constituted a core structure of MCM2-7 hexamers (Champasa et al., 2019). We found that MCM4 played a core role in all analyses (using ONCOMINE, GEPIA, PROGNOSIS biomarker and KEGG datasets) and the differential expression of MCM4 in osteosarcoma cell line was confirmed using qRT-PCR and western blot, this has led us to further explore the mechanism of prognostic value of MCM4 in sarcoma.

### Methylation of MCM4 Promoter Inhibits the Transactivation of Potential Transcription Factors

**FIGURE 6:**
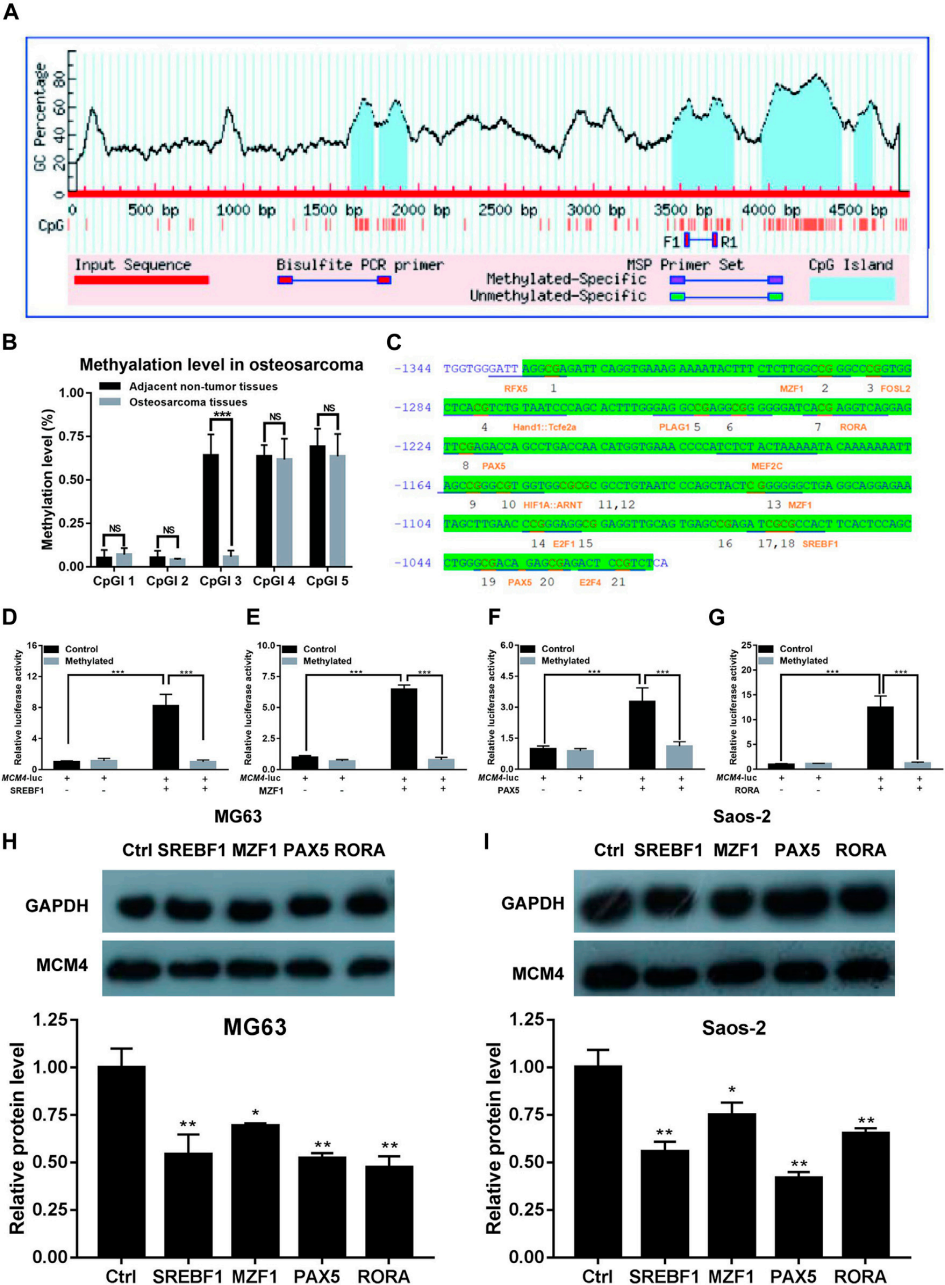
Methylation analysis of MCM4. **(A)** Five CpG islands were predicted in the MCM4 promoter region. **(B)** The methylation rate of CpGI3 was significantly decreased in osteosarcoma tissues compared with the normal tumor-adjacent lung tissues. **(C)** A large CpG island predicted in the MCM4 promoter. **(D–G)** SREBF1, MZF1, PAX5, and RORA candidate transcription factor transactivation of MCM4 expression in cell transfection experiment. Knockdown of SREBF1, MZF1, PAX5, and RORA would reduce the MCM4 protein level in MG63 **(H)** and Saos-2 **(I)** cell lines, *p < 0.05, **p < 0.01, ***p < 0.001.

We obtained the 5 kb MCM4 promoter sequence via the Ensembl genome browser. CpG islands were identified using MethPrimer software. Five CpG islands were identified in the 5 kb promoter region (Figure 6A). The degree of methylation of CpGI three was significantly reduced in patients with osteosarcoma (Figure 6B). To check MCM4 gene modulation, the transcription factor binding sites of the CpGI three were analyzed using JASPAR. In the CpGI 3, the following transcription factors were predicted to interact with the CpG sites: RFX5, M2F1, FOSL2, PLAG1, RORA, PAX5, MEF2C, MZF1, E2F1, SREBF1, PAX5, and E2F4 (Figure 6C). We selected these four transcription factors with the highest scores including SREBF1, MZF1, PAX5, and RORA for further investigation. According to the results, we found that transcription factors SREBF1, MZF1, PAX5, and RORA activated MCM4-luc expression, but transcription factors SREBF1, MZF1, PAX5 and RORA were not able to activate MCM4-luc expression when MCM4-luc was methylated *in vivo* (Figures 6D–G). Additionally, we found that knockdown of these transcription factors including SREBF1, MZF1, PAX5 and RORA can reduce the MCM4 protein level in MG63 and Saos-2 cell line (Figures 6H,I). The results indicated that transactivation of potential transcription factors is affected by methylated MCM4 promoter.

### Demethylation of MCM4 on the Proliferation and Apoptosis of hFOB1.19 Cells

**FIGURE 7:**
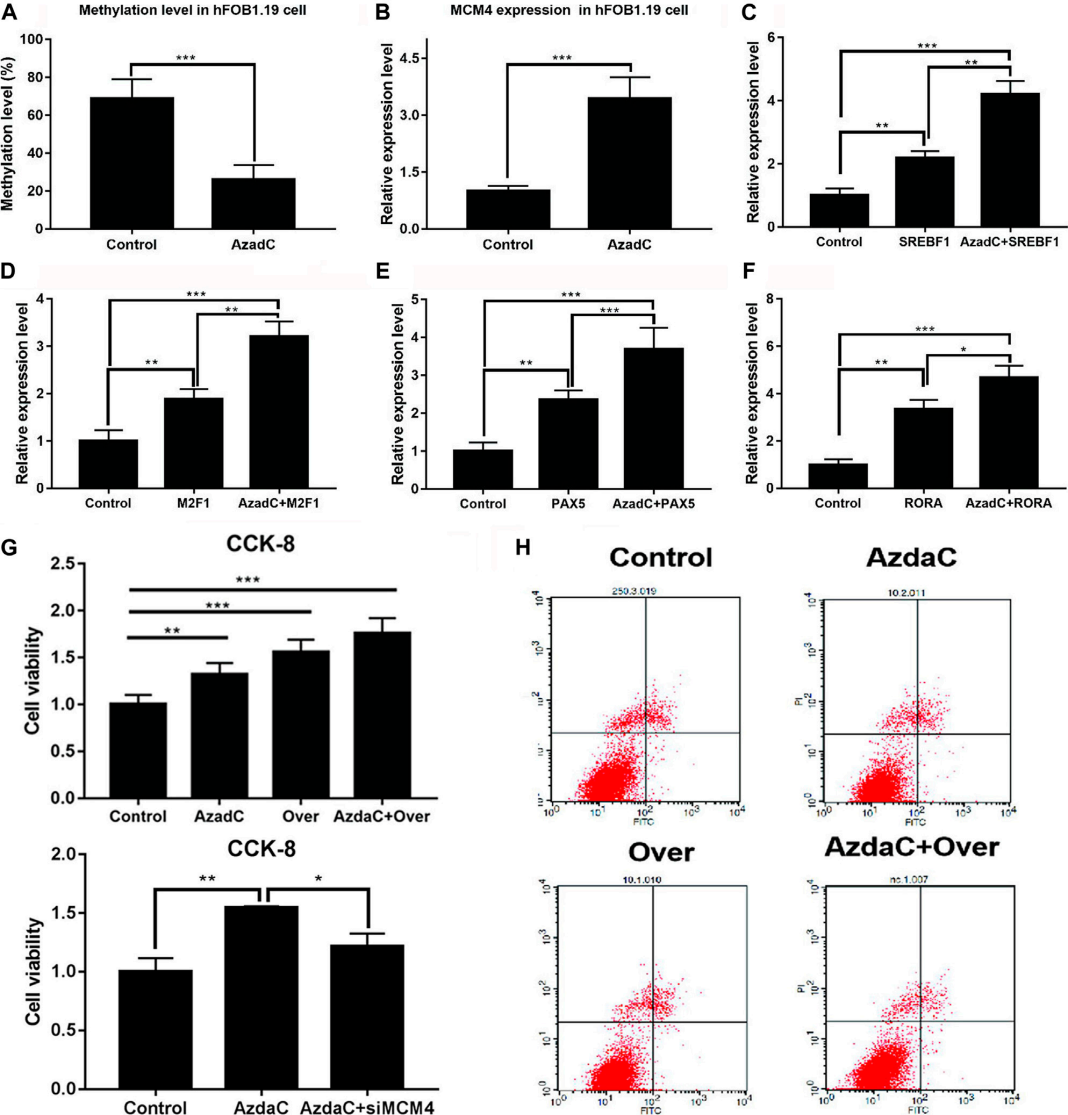
Cell proliferation and apoptosis analysis of MCM4 demethylation and overexpression. **(A)** After treatment with AzadC, MCM4 promoter methylation level showed a significantly decreased methylation rate. **(B)** MCM4 mRNA level significantly increased. **(C–F)** The activity of transcription factors (SREBF1, MZF1, PAX5, and RORA) increased. **(G)** AzadC treatment and overexpression of MCM4 significantly promoted hFOB1.19 cell proliferation. SiMCM4 can decrease the effect of hFOB1.19 cell proliferation induced by AzadC. **(H)** AzadC treatment and overexpression of MCM4 significantly inhibited hFOB1.19 cell apoptosis, *p < 0.05, **p < 0.01, ***p < 0.001.

Bisulfite sequencing PCR results indicated that the methylation ratio of the MCM4 promoter decreased after AzadC treatment (Figure 7A). The results of qRT-PCR showed that expression of MCM4 significantly increased after AzadC treatment (Figure 7B). We investigated if overexpression of potential transcription factors increased demethylated MCM4 gene expression. HFOB1.19 cells were co-transfected with potential transcription factors. The results of qRT-PCR indicated that the transcription factors SREBF1, MZF1, PAX5, and RORA could activate MCM4 expression alone. When SREBF1, MZF1, PAX5, and RORA were treated along with AzadC, the expression of MCM4 expression increased significantly, compared with that observed for transcription factor alone (Figures 7C–F).

Moreover, the CCK-8 and TUNEL experiment was conducted to detect hFOB1.19 cellular proliferation and apoptosis after treatment with AzadC. The results indicated that AzadC promoted hFOB1.19 proliferation compared to control. In order to check the MCM4 function, overexpression was performed and the results indicated that sole overexpression of MCM4 obtained similar results as AzadC treatment. Together, MCM4 overexpression and AzadC treatment showed the stronger promotion of cell proliferation, and siMCM4 decreased the effect of proliferation induced by AzadC (Figure 7G). The TUNEL experiment results showed that AzadC decreased the apoptosis of hFOB1.19 cells compared to control. The sole overexpression of MCM4 obtained similar results as AzadC treatment. Together, MCM4 overexpression and AzadC treatment showed stronger inhibition of cell apoptosis than the control (Figure 7H). The results showed that decreased MCM4 methylation enhanced cellular proliferation and inhibited apoptosis in hFOB1.19 cells.

## Discussion

Proteins involved in the replication of DNA were widely proposed as promising cancer biomarkers (Yu et al., 2020). MCMs family has ten members and each of them was essential for viability. This protein family plays an important role in different stages of DNA replication, especially the initial step (Edwards et al., 2002; Das and Rhind, 2016). The overexpression of MCM factors was identified in multiple cancers, including breast cancer, lung cancer and colorectal cancer (Gonzalez et al., 2003; Nishihara et al., 2008; Liu et al., 2017; Yu et al., 2020). To our best knowledge, this is the first study that systematically analyzed the expression and prognostic value of MCM factors in human sarcoma. The study results may have important implications for improving the prognosis of sarcoma patients.

MCM1 was reported to be localized at the replication origins of DNA and influences the local structure of replication origins (Chang et al., 2003). In our study, we analyzed the expression level of MCM1 suing ONCOMINE datasets, and found that the expression level of MCM1 was higher in sarcoma tissue than in normal tissue. But in GEPIA datasets, the result was opposite. Then we searched the expression levels of MCM1 in the cell lines by using CCLE datasets and found MCM1 was highly expressed in human cell lines. With GEPIA datasets, we tried to explore the prognostic value of MCM1 in sarcoma patients, but the result showed that there was no significant relationship between the expression level of MCM1 and DFS or OS of patients with sarcoma.

MCM2-7 protein complex exhibits DNA helicase activity and plays central roles in regulating transcription, chromatin remodeling and checkpoint responses (Ishimi, 2018). Previous studies demonstrated that MCM2-7 protein complex could act as biomarkers for dysplasia and malignancy (Freeman et al., 1999). It was also showed to be prognostic markers for many kinds of human cancers (Liu et al., 2017). We analyzed the expression levels of these six genes in ONCOMINE datasets and GEPIA datasets, the results showed that they were all upregulated in sarcoma compared with normal tissues. Using CCLE datasets, we analyzed their expression levels in sarcoma cell lines and found that they were all highly expressed. But the prognosis value of the six genes in sarcoma was different. With further using GEPIA datasets, we analyzed the association between the high expression of these genes and the OS and DSF of sarcoma patients. The results showed that high expression of MCM2, MCM3 and MCM4 was significantly related to poor OS of sarcoma patients. Highly expressed MCM3 was also significantly related to poor DFS of sarcoma patients. The other three genes had no significant relation between the expression levels and the prognosis of sarcoma patients. So MCM2, MCM3, and MCM4 seemed to be three potential biomarkers for the prognosis of sarcoma.

MCM8–9 also formed a complex and was a homolog of the MCM2–7 hetero-hexameric helicase complex. The resent studies claimed that MCM8–9 played an essential role during replication elongation and recombination of DNA (Maiorano et al., 2005; Gambus and Blow, 2013). Cancer cells underwent more replication stress because they were hyperstimulated to grow, and it was reported that inhibiting MCM8-9 could increase the sensitivity of tumors to cisplatin (Morii et al., 2019). In the present study, we analyzed the expression levels of MCM8 and MCM9 in ONCOMINE datasets. But there was no data about the two factors. Then we searched their expression level in GEPIA datasets, and the results showed that both of them were upregulated in sarcoma compared to normal tissues. Using CCLE datasets, we found that MCM8 and MCM9 were both overexpressed in che cell line of sarcoma. At last, we analyzed the association between expression levels of the two genes and the OS and DFS of sarcoma patients, with no significant associations observed.

MCM10, an important regulator of DNA replication initiation, was found to be crucial to maintain genome integrity (Bielinsky, 2016). There is accumulating evidence suggesting that in the development of tumor, dysregulation of MCM10 contributed to aberrant proliferation and genome instability. MCM10 was reported to play an important role in several tumors including breast cancer and urothelial carcinoma (Li et al., 2016; Yang and Wang, 2019). In our study, we analyzed the expression level of MCM10 in ONCOMINE datasets and GEPIA datasets. The results showed that MCM10 was upregulated in sarcoma compared to normal samples. In CCLE, we also found that MCM10 was highly expressed in sarcoma cell lines. To explore the prognosis value of MCM10 in sarcoma, we analyzed the data in GEPIA and found that highly expressed MCM10 was associated with poor OS of sarcoma patients, indicating that MCM10 was a potential biomarker of prognosis for sarcoma patients.

According to our results, we also found that MCM4 played a core role in ONCOMINE, GEPIA, PROGNOSIS biomarker and KEGG analysis and the differential expression of MCM4 in osteosarcoma cell line was confirmed using qRT-PCR and western blot. Therefore, the mechanism of prognostic value of MCM4 in sarcoma was explored. The results revealed that demethylation treatment increased the transactivation of potential transcription factors and enabled high levels of MCM4 expression in hFOB1.19 cells. CKK-8 and TUNEL experiment was conducted and the results showed that decreased MCM4 methylation enhanced cellular proliferation and inhibited apoptosis in hFOB1.19 cells. Therefore, the prognostic role of MCM4 in sarcoma may be attributable to changes in DNA methylation patterns. There was limitation in the present study. The data used for analysis were obtained from online services. We need to carry out more clinical experiments in a well-established tumor cohort to confirm our findings.

## Conclusions

In this study, we found that MCM2, 3, 4, and 10 could be used as molecular markers to identify high-risk subgroups of sarcoma patients. The four MCM family members, MCM2, 3, 4, and 10 could be prognostic biomarkers for human sarcoma and a higher expression of these MCM factors predicts poorer outcomes. The prognostic role of MCM4 may be attributable to changes in DNA methylation patterns.

## Data Availability

The original contributions presented in the study are included in the article/Supplementary Material, further inquiries can be directed to the corresponding authors.The datasets used and/or analyzed during the current study are available from the corresponding author on reasonable request.

## References

[B1] Barretina J. Taylor B. S. Banerji S. Ramos A. H. Lagos-Quintana M. DeCarolis P. L. (2010). Subtype-specific Genomic Alterations Define New Targets for Soft-Tissue Sarcoma Therapy. Nat. Genet. 42, 715–721. 10.1038/ng.619 20601955 PMC2911503

[B2] Baxley R. Bielinsky A.-K. (2017). Mcm10: A Dynamic Scaffold at Eukaryotic Replication forks. Genes 8, 73. 10.3390/genes8020073 PMC5333062 28218679

[B3] Bielinsky A.-K. (2016). Mcm10: The Glue at Replication forks. Cell Cycle 15, 3024–3025. 10.1080/15384101.2016.1216925 27485176 PMC5134715

[B4] Cai H.-Q. Cheng Z.-J. Zhang H.-P. Wang P.-F. Zhang Y. Hao J.-J. (2018). Overexpression of MCM6 Predicts Poor Survival in Patients with Glioma. Hum. Pathol. 78, 182–187. 10.1016/j.humpath.2018.04.024 29753008

[B5] Champasa K. Blank C. Friedman L. J. Gelles J. Bell S. P. (2019). A Conserved Mcm4 Motif Is Required for Mcm2-7 Double-Hexamer Formation and Origin DNA Unwinding. Elife 8. e45538. 10.7554/eLife.45538 31385807 PMC6701924

[B6] Chang V. K. Fitch M. J. Donato J. J. Christensen T. W. Merchant A. M. Tye B. K. (2003). Mcm1 Binds Replication Origins. J. Biol. Chem. 278, 6093–6100. 10.1074/jbc.M209827200 12473677

[B7] Dancsok A. R. Asleh-Aburaya K. Nielsen T. O. (2017). Advances in Sarcoma Diagnostics and Treatment. Oncotarget 8, 7068–7093. 10.18632/oncotarget.12548 27732970 PMC5351692

[B8] Das S. P. Rhind N. (2016). How and Why Multiple MCMs Are Loaded at Origins of DNA Replication. Bioessays 38, 613–617. 10.1002/bies.201600012 27174869 PMC5052224

[B9] Detwiller K. Y. Fernando N. T. Segal N. H. Ryeom S. W. D'Amore P. A. Yoon S. S. (2005). Analysis of Hypoxia-Related Gene Expression in Sarcomas and Effect of Hypoxia on RNA Interference of Vascular Endothelial Cell Growth Factor a. Cancer Res. 65, 5881–5889. 10.1158/0008-5472.CAN-04-4078 15994966

[B10] Edwards M. C. Tutter A. V. Cvetic C. Gilbert C. H. Prokhorova T. A. Walter J. C. (2002). MCM2-7 Complexes Bind Chromatin in a Distributed Pattern Surrounding the Origin Recognition Complex inXenopus Egg Extracts. J. Biol. Chem. 277, 33049–33057. 10.1074/jbc.M204438200 12087101

[B11] Fei L. Xu H. (2018). Role of MCM2-7 Protein Phosphorylation in Human Cancer Cells. Cell Biosci. 8, 43. 10.1186/s13578-018-0242-2 30062004 PMC6056998

[B12] Freeman A. Morris L. S. Mills A. D. Stoeber K. Laskey R. A. Williams G. H. (1999). Minichromosome Maintenance Proteins as Biological Markers of Dysplasia and Malignancy. Clin. Cancer Res. 5, 2121–2132. 10473096

[B13] Gambus A. Blow J. J. (2013). Mcm8 and Mcm9 Form a Dimeric Complex inXenopus Laevisegg Extract that Is Not Essential for DNA Replication Initiation. Cell Cycle 12, 1225–1232. 10.4161/cc.24310 23518502 PMC3674087

[B14] Giaginis C. Georgiadou M. Dimakopoulou K. Tsourouflis G. Gatzidou E. Kouraklis G. (2009). Clinical Significance of MCM-2 and MCM-5 Expression in colon Cancer: Association with Clinicopathological Parameters and Tumor Proliferative Capacity. Dig. Dis. Sci. 54, 282–291. 10.1007/s10620-008-0305-z 18465232

[B15] Gonzalez M. A. Pinder S. E. Callagy G. Vowler S. L. Morris L. S. Bird K. (2003). Minichromosome Maintenance Protein 2 Is a strong Independent Prognostic Marker in Breast Cancer. J. Clin. Oncol. 21, 4306–4313. 10.1200/JCO.2003.04.121 14645419

[B16] Griffin W. C. Trakselis M. A. (2019). The MCM8/9 Complex: A Recent Recruit to the Roster of Helicases Involved in Genome Maintenance. DNA Repair 76, 1–10. 10.1016/j.dnarep.2019.02.003 30743181 PMC9202240

[B17] Ishimi Y. (2018). Regulation of MCM2-7 Function. Genes Genet. Syst. 93, 125–133. 10.1266/ggs.18-00026 30369561

[B18] Li S. Jiang Z. Li Y. Xu Y. (2019). Prognostic Significance of Minichromosome Maintenance mRNA Expression in Human Lung Adenocarcinoma. Oncol. Rep. 42, 2279–2292. 10.3892/or.2019.7330 31545501 PMC6826304

[B19] Li W.-M. Huang C.-N. Ke H.-L. Li C.-C. Wei Y.-C. Yeh H.-C. (2016). MCM10 Overexpression Implicates Adverse Prognosis in Urothelial Carcinoma. Oncotarget 7, 77777–77792. 10.18632/oncotarget.12795 27780919 PMC5363620

[B20] Liu Y.-Z. Wang B.-S. Jiang Y.-Y. Cao J. Hao J.-J. Zhang Y. (2017). MCMs Expression in Lung Cancer: Implication of Prognostic Significance. J. Cancer 8, 3641–3647. 10.7150/jca.20777 29151950 PMC5688916

[B21] Maiorano D. Cuvier O. Danis E. Méchali M. (2005). MCM8 Is an MCM2-7-Related Protein that Functions as a DNA Helicase during Replication Elongation and Not Initiation. Cell 120, 315–328. 10.1016/j.cell.2004.12.010 15707891

[B22] Maiorano D. Lutzmann M. Méchali M. (2006). MCM Proteins and DNA Replication. Curr. Opin. Cel Biol. 18, 130–136. 10.1016/j.ceb.2006.02.006 16495042

[B23] Marnerides A. Vassilakopoulos T. P. Boltetsou E. Levidou G. Angelopoulou M. K. Thymara I. (2011). Immunohistochemical Expression and Prognostic Significance of CCND3, MCM2 and MCM7 in Hodgkin Lymhoma. Anticancer Res. 31, 3585–3594. 21965782

[B24] Morii I. Iwabuchi Y. Mori S. Suekuni M. Natsume T. Yoshida K. (2019). Inhibiting the MCM8-9 Complex Selectively Sensitizes Cancer Cells to Cisplatin and Olaparib. Cancer Sci. 110, 1044–1053. 10.1111/cas.13941 30648820 PMC6398883

[B25] Nishihara K. Shomori K. Fujioka S. Tokuyasu N. Inaba A. Osaki M. (2008). Minichromosome Maintenance Protein 7 in Colorectal Cancer: Implication of Prognostic Significance. Int. J. Oncol. 33, 245–251. 10.3892/ijo_00000003 18636144

[B26] Peng Y.-P. Zhu Y. Yin L.-D. Zhang J.-J. Guo S. Fu Y. (2016). The Expression and Prognostic Roles of MCMs in Pancreatic Cancer. PLoS One 11, e0164150. 10.1371/journal.pone.0164150 27695057 PMC5047525

[B27] Pramila T. Miles S. GuhaThakurta D. Jemiolo D. Breeden L. L. (2002). Conserved Homeodomain Proteins Interact with MADS Box Protein Mcm1 to Restrict ECB-dependent Transcription to the M/G1 Phase of the Cell Cycle. Genes Dev. 16, 3034–3045. 10.1101/gad.1034302 12464633 PMC187489

[B28] Quade B. J. Wang T.-Y. Sornberger K. Cin P. D. Mutter G. L. Morton C. C. (2004). Molecular Pathogenesis of Uterine Smooth Muscle Tumors from Transcriptional Profiling. Genes Chromosom. Cancer 40, 97–108. 10.1002/gcc.20018 15101043

[B29] Rusiniak M. E. Kunnev D. Freeland A. Cady G. K. Pruitt S. C. (2012). Mcm2 Deficiency Results in Short Deletions Allowing High Resolution Identification of Genes Contributing to Lymphoblastic Lymphoma. Oncogene 31, 4034–4044. 10.1038/onc.2011.566 22158038 PMC3309111

[B30] Shomori K. Nishihara K. Tamura T. Tatebe S. Horie Y. Nosaka K. (2010). Geminin, Ki67, and Minichromosome Maintenance 2 in Gastric Hyperplastic Polyps, Adenomas, and Intestinal-type Carcinomas: Pathobiological Significance. Gastric Cancer 13, 177–185. 10.1007/s10120-010-0558-z 20820987

[B31] Stewart P. A. Khamis Z. I. Zhau H. E. Duan P. Li Q. Chung L. W. K. (2017). Upregulation of Minichromosome Maintenance Complex Component 3 during Epithelial-To-Mesenchymal Transition in Human Prostate Cancer. Oncotarget 8, 39209–39217. 10.18632/oncotarget.16835 28424404 PMC5503607

[B32] Suzuki Y. Yamaguchi Y. Hanada H. Ishimi Y. (2019). Changes in MCM2-7 Proteins at Senescence. Genes Genet. Syst. 94, 123–132. 10.1266/ggs.18-00062 31092751

[B33] Winther T. L. Torp S. H. (2017). MCM7 Expression Is a Promising Predictor of Recurrence in Patients Surgically Resected for Meningiomas. J. Neurooncol. 131, 575–583. 10.1007/s11060-016-2329-0 27868157

[B34] Wojnar A. Pula B. Piotrowska A. Jethon A. Kujawa K. Kobierzycki C. (2011). Correlation of Intensity of MT-I/II Expression with Ki-67 and MCM-2 Proteins in Invasive Ductal Breast Carcinoma. Anticancer Res. 31, 3027–3033. 21868554

[B35] Wu W. Wang X. Shan C. Li Y. Li F. (2018). Minichromosome Maintenance Protein 2 Correlates with the Malignant Status and Regulates Proliferation and Cell Cycle in Lung Squamous Cell Carcinoma. Onco. Targets Ther. 11, 5025–5034. 10.2147/OTT.S169002 30174440 PMC6109654

[B36] Yang W. D. Wang L. (2019). MCM10 Facilitates the Invaded/migrated Potentials of Breast Cancer Cells via Wnt/β‐catenin Signaling and Is Positively Interlinked with Poor Prognosis in Breast Carcinoma. J. Biochem. Mol. Toxicol. 33, e22330. 10.1002/jbt.22330 30990947

[B37] Yu S. Wang G. Shi Y. Xu H. Zheng Y. Chen Y. (2020). MCMs in Cancer: Prognostic Potential and Mechanisms. Anal. Cell Pathol. 2020, 1–11. 10.1155/2020/3750294 PMC7023756 32089988

[B38] Zhong H. Chen B. Neves H. Xing J. Ye Y. Lin Y. (2017). Expression of Minichromosome Maintenance Genes in Renal Cell Carcinoma. Cancer Manag. Res. 9, 637–647. 10.2147/CMAR.S146528 29180899 PMC5697450

